# Supplementary figures and images for: Co-inhibition of adenosine 2b receptor and programmed death-ligand 1 promotes the recruitment and cytotoxicity of natural killer cells in oral squamous cell carcinoma
Source: PeerJ. 2023 Aug 30;11:e15922. doi: 10.7717/peerj.15922 (PMC10474825; doi:10.7717/peerj.15922)

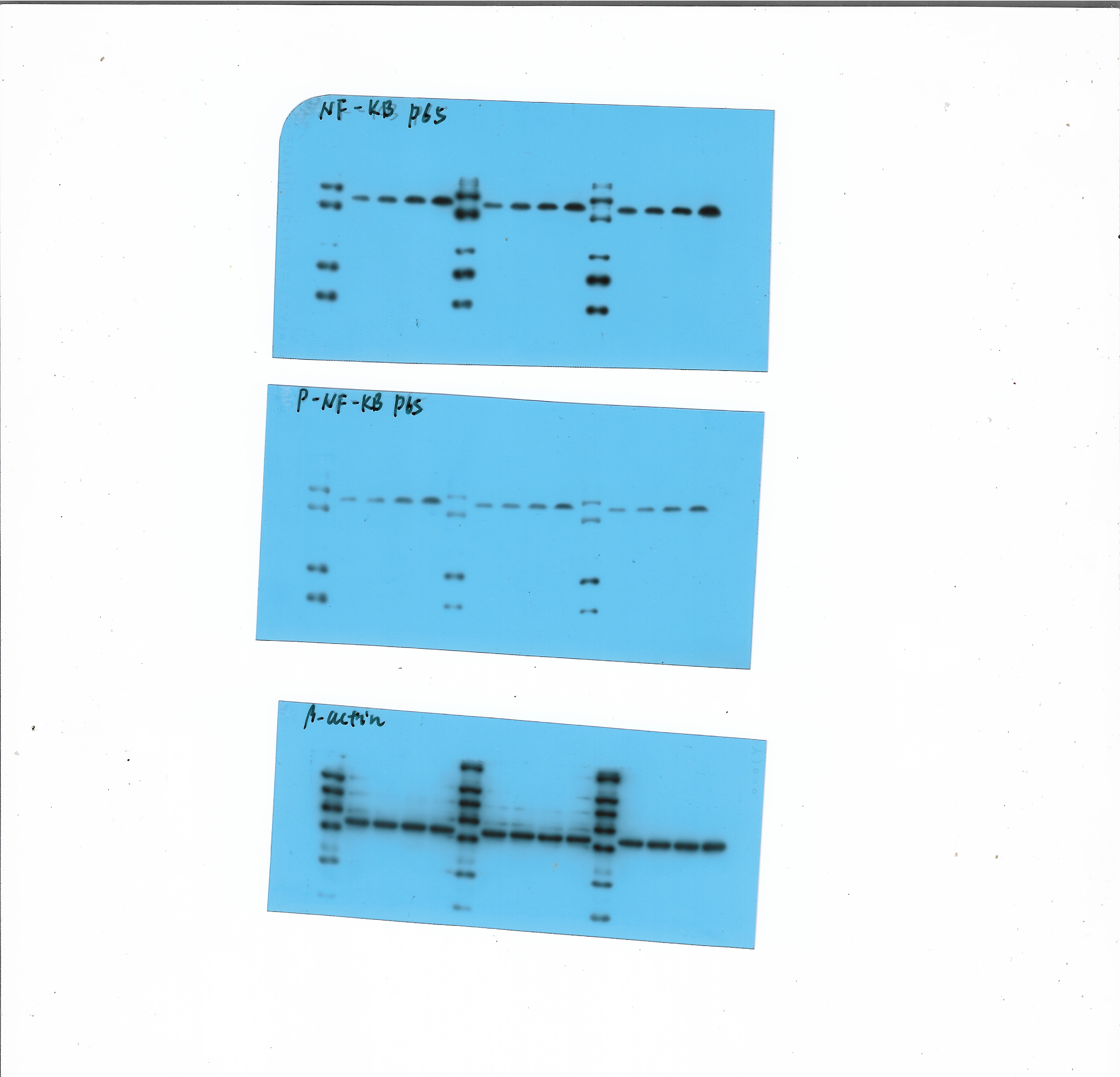

Supplement: Supplemental Information 1 [file peerj-11-15922-s001.jpg]
